# *Hafnia alvei* Urosepsis in a Kidney Transplant Patient

**DOI:** 10.1155/2015/863131

**Published:** 2015-04-15

**Authors:** Mario Stanic, Edgar Meusburger, Gabriele Hartmann, Karl Lhotta

**Affiliations:** ^1^Department of Nephrology and Dialysis, Academic Teaching Hospital Feldkirch, 6800 Feldkirch, Austria; ^2^Institute for Pathology, Academic Teaching Hospital Feldkirch, 6800 Feldkirch, Austria

## Abstract

*Hafnia alvei*, a gram-negative facultative anaerobic, rod-shaped bacterium, is a rare cause of infection in humans. We report on a renal transplant patient who developed *H. alvei* pyelonephritis and urosepsis. The source of infection remains enigmatic but is most likely the intestinal tract. Appropriate antibiotic therapy with cefepime followed by oral ciprofloxacin brought about rapid resolution of symptoms and complete recovery. *H. alvei* may cause severe infection in transplant patients without predisposing factors such as hospitalization, invasive procedures, or antibiotic treatment.

## 1. Introduction

*Hafnia alvei* is a facultative anaerobic gram-negative bacterium belonging to the Enterobacteriaceae family. Although this genus was first described in 1954, very little is known about its role in human infectious diseases.* H. alvei* can be recovered from soil, water, and many animal species, especially mammals, where it colonises primarily the gastrointestinal tract [1]. In humans,* H. alvei* seems to be a member of the normal gut microbiome as a nonpathogenic inhabitant, although some studies conclude that these bacteria cause significant clinical gastroenteritis [1, 2], especially in children [3], but also in adult patients suffering from chronic disease or after previous antibiotic therapy [4]. It would appear that* H. alvei* is a microorganism with limited pathogenicity in humans that may cause clinically significant infections only in immunocompromised individuals, for example, those with malignancy or after organ transplantation [5, 6]. We here describe a renal transplant recipient who suffered from* H. alvei* urosepsis.

## 2. Case Report

The 56-year-old woman developed end-stage renal disease due to IgA nephropathy. In August 2012 she underwent preemptive kidney transplantation, receiving a kidney allograft from her husband. Her posttransplant course was stable with a serum creatinine of 0.90 mg/dL (eGFR 75 mL/min), and medical therapy consisted of tacrolimus (blood levels between 5 and 7 ng/mL), azathioprine, simvastatin, and pantoprazole.

Two days before admission in November 2014 she experienced headache, shivering, nausea, repeated vomiting, and diarrhea. At admission she had fever of 38°C. Blood tests showed a mild leukocytosis of 10.5 G/L (neutrophils 84%) and CRP of 20 mg/dL and her serum creatinine had increased to 1.30 mg/dL. An ultrasound of the transplant was unremarkable. The urinary sediment contained multiple leukocytes and bacteria. A diagnosis of transplant pyelonephritis was made and cefepime 2 g b.i.d. was commenced. Cultures taken from urine and blood samples taken on admission grew* H. alvei* identified by matrix-assisted laser desorption ionisation time-of-flight mass spectrometry (MALDI TOF) (MALDI Biotyper©Microflex LT, Bruker Daltonics, Bremen, Germany). No coinfection with other bacteria could be identified. The results of antimicrobial susceptibility testing using the VITEK 2 system (bioMerieux) are shown in Table 1.

Under antibiotic therapy the patient's symptoms and fever subsided and serum creatinine decreased to 0.90 mg/dL within two days. After five days antibiotic therapy was switched to ciprofloxacin 500 mg b.i.d. and the patient was discharged. At an outpatient control one week later she was in excellent clinical condition, laboratory parameters had normalised, and antibiotic therapy could be discontinued.

## 3. Discussion

Although relatively little is known about the role of* H. alvei* in human clinical infection, some common themes do emerge. First of all,* H. alvei* is rarely found in human specimens, mostly from the respiratory and gastrointestinal tract, urine, blood, wounds, and abscesses [9, 10]. In the majority of these cases, however, the bacterium does not seem to be related to the clinical infection. For example, whether* H. alvei* can cause acute gastroenteritis is still a matter of debate [1, 2]. Günthard and Pennekamp found* H. alvei* in 80 specimens but considered it to be pathogenetic in only three patients (two with septicemia and one with peritonitis). Secondly, most patients seem to have underlying conditions which predispose them to infection. For example, in the series studied by Günthard and Pennekamp, 93% of the patients had an underlying disease, mainly malignancy. Thirdly, in most cases infection with* H. alvei* is nosocomial. Rodríguez-Guardado in a collective of 36 patients with extraintestinal infection due to* H. alvei* over an eleven-year period found that 25 were nosocomial and eleven community-acquired [10]. In addition to underlying comorbidities, 76% of patients had other predisposing risk factors such as surgery, intravenous catheter insertion, or antibiotic therapy [9–11]. And lastly, in most specimens a coinfection with other, more pathogenic bacteria such as* Staphylococcus aureus* was seen [10].

At our institution 40* H. alvei* isolates have been detected over the last ten years. Most positive specimens were obtained from respiratory tract secretions in intubated patients or from infections after abdominal surgery. Only three positive blood cultures were found and two of these patients had an underlying malignancy.

Several cases of* H. alvei* infection have been described in stem cell and particularly solid organ transplant recipients. Intestinal colonization and possible infection with* H. alvei* have been reported in a 9-year-old girl after hematopoietic stem cell transplantation for Fanconi's anemia. Two patients, one 61-year-old woman and a 2-year-old boy, developed hepatic abscess after liver transplantation. The first patient had coinfection with* Enterococcus faecalis* and* Candida albicans* and the second with* Enterococcus faecalis*. Immunosuppressive and antimicrobial treatment are specified in Table 2. Günthard and Pennekamp reported a positive specimen in a lung transplant patient, but without further details.

Clinically significant* H. alvei* infection has also been reported in two renal transplant recipients.

The first patient was a 69-year-old female who developed sepsis and pneumonia nine years after renal transplantation. Basal immunosuppression consisted of cyclosporine, azathioprine, and steroids. The patient also suffered from hepatitis C infection. She developed hypotension, oliguria, and coagulopathy and recovered after hemodynamic support and antibiotic therapy with imipenem and clarithromycin.

The second case was a 45-year-old female suffering from ESRD due to diabetic nephropathy. Her course after transplantation was complicated by acute humoral rejection and chronic rejection.* H. alvei* pyelonephritis developed after ureteral stent implantation for hydroureteronephrosis. The patient recovered after treatment with ceftriaxone, cefixime, and removal of the stent.

Our case is similar to these two reports. We also were unable to identify the source of the infection. Certain foods, especially meat and fish, may harbour* H. alvei* in large amounts [12]. As* H. alvei* is part of the normal gut microbiota, we believe that the gastrointestinal tract, as common in urinary tract infections, was the most likely source of infection. None of these kidney transplant patients had a coinfection with other more aggressive bacteria. The two other patients, however, had additional comorbidities, namely, diabetes in one and hepatitis C infection in the other patient. In one patient* H. alvei* infection developed after an invasive procedure. The other two patients had triple immunosuppressive therapy including prednisolone, whereas our patient only had double therapy without steroids. All three patients ultimately recovered after appropriate antibiotic therapy.

In conclusion,* H. alvei* can cause severe infection such as urosepsis, pyelonephritis, and pneumonia in renal allograft recipients. Antimicrobial therapy with carbapenems, quinolones, or fourth-generation cephalosporins is necessary to cure the infection.

## Figures and Tables

**Table 1:** Susceptibility testing of *H. alvei* recovered from the blood culture.

Sensitive	Resistant
Cefotaxime/ceftazidime	Ampicillin
Cefepime	Amoxicillin/clavulanic acid
Ertapenem/meropenem	Piperacillin/tazobactam
Gentamicin	Fosfomycin
Ciprofloxacin	
Trimethoprim/sulfamethoxazole	

**Table 2:** *H. alvei* infections in transplant recipients.

Author	Age	Sex	Underlying disease	Type of transplant	Immunosuppression	Risk factors	Antibiotic treatment	Outcome
Savini et al. [5]	9	F	Fanconi's anaemia	Allogeneic stem cells	Cyclosporine steroids antilymphocyte antibody	Graft versus host disease	Ceftazidime, amikacin, and teicoplanin	Cured

Barry et al. [6]	61	F	Primary biliary cirrhosis	Liver	Unknown	Recurrent rejection, additional infection	Piperacillin/ tazobactam, gentamicin, and amphotericin B	Died

Barry et al. [6]	2	M	Autoimmune hepatitis	Liver	Unknown	Previous high dose steroids and OKT3 therapy	Trimethoprim/ sulfamethoxazole, gentamicin, and ampicillin	Cured

Cardile et al. [7]	45	F	Diabetic nephropathy	Kidney	Tacrolimus MMF prednisone	Diabetes ureteral stent	Cefixime	Cured

Benito et al. [8]	69	F	Autosomal dominant polycystic kidney disease	Kidney	Cyclosporine azathioprine steroids	Hepatitis C	Imipenem and clarithromycin	Cured

This study	56	F	IgA nephropathy	Kidney	Tacrolimus azathioprine	None	Cefepime and ciprofloxacin	Cured
